# A practical approach to the management of breast cancer in the COVID-19 era and beyond

**DOI:** 10.3332/ecancer.2020.1059

**Published:** 2020-06-17

**Authors:** Alison Luther, Avi Agrawal

**Affiliations:** Breast Services, Queen Alexandra Hospital, Portsmouth PO6 3LY, UK

**Keywords:** breast cancer, COVID-19

## Abstract

The COVID-19 pandemic has resulted in a major shift in how breast services are being utilised and managed. The guidelines relating to this have been published by recognised medical associations from the UK, Europe and the USA, addressing many aspects of breast cancer care. There is an awareness that there may be limitations in resources during this time, and therefore, prioritisation strategies have been identified to ensure that breast cancer patients are appropriately treated, whilst reducing the risk of infection from COVID-19 to both patients and staff.

Equally important is the consideration of how breast cancer services can be safely re-introduced, as infection rates from COVID-19 decline. However, until there is a definite management strategy for COVID-19, such as a vaccine being developed, it is likely that there will still be a significant impact from COVID-19 on breast cancer care. This paper, therefore, aims to highlight the current guidance and evidence regarding breast cancer management in the era of COVID-19, and also aims to look at future management strategies in this period of uncertainty.

## Introduction

The first documented cases of COVID-19 were identified in the Wuhan province, China, in December 2019, and there are now currently over 5 million cases confirmed worldwide [1–3]. COVID-19 is caused by the severe acute respiratory coronavirus 2 (SARS-CoV-2), and may present with a constellation of symptoms, including pyrexia, cough and respiratory distress [2, 4]. It is approximately twice as infective as the seasonal influenza virus with an *R*_0_ of 2.2 (average number of patients infected from index patient) [2, 5, 6]. This is felt to be due to the long incubation period of COVID-19, where individuals can be asymptomatic for up to 14 days, yet still have potential to transmit the disease to others. The SARS-CoV-2 virus is primarily spread in large respiratory droplets but has also been detected in both blood and stool [5, 7–9].

The mortality rate from COVID-19 has been challenging to assess, as for many countries, testing has been limited to patients presenting with severe symptoms, or health care workers. Estimates of the mortality rate however range from 0.1% to 5%. Certain subsets of the population, however, i.e., the elderly and those with underlying co-morbidities are at much higher risk, and many countries have employed ‘shielding’ methods to protect the most vulnerable [1, 2, 5, 6,8, 10].

It is currently impossible to be working in any field of medicine without being affected by the COVID-19 pandemic. Whilst breast surgeons and associated multidisciplinary team (MDT) members may not be directly managing the unwell COVID-19 patient, the consequences of the increased demand on staffing, both from sickness and redeployment, are likely to impact services. From an operating perspective, both theatre space and ventilators may be in demand for overspill from intensive care units, with anaesthetists being required to oversee this. Prioritisation of patients, and alternative assessment and management strategies must therefore be considered and implemented to minimise risk of infection to both staff and patients. Even in regions where the rates of COVID-19 are in decline, until the introduction of a viable management strategy, such as a vaccine, concepts such as social distancing, and concerns regarding a ‘second peak’ of COVID-19, will continue to affect breast cancer care in many ways. This paper, therefore, aims to look at all parts of the breast cancer patient pathway in the context of COVID-19, with up to date evidence and guidelines. It also aims to look at how breast cancer services can be safely reintroduced in units where the burden of COVID-19 is decreasing, whilst maintaining the safety of both patients and health care providers.

## Current guidelines

**Figure 1.**
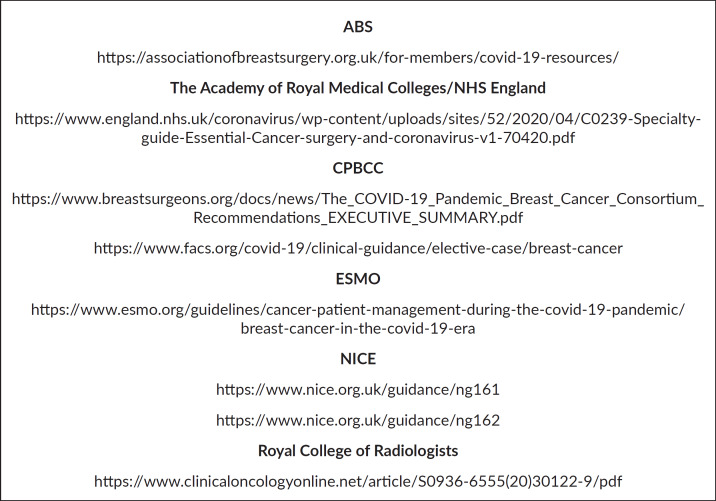
Links to key guidelines [11–17].

The Association of Breast Surgeons (ABS), the Royal College of Radiologists, the Academy of Medical Royal Colleges, the COVID-19 Pandemic Breast Cancer Consortium (CPBCC), which includes the American Society of Breast Surgeons and the American Association of Surgeons, the European Society for Medical Oncology (ESMO) and the National Institute for Clinical Excellence (NICE) have all issued new guidance pertaining to aspects of breast cancer care during this pandemic. Links to all of these are included in Figure 1 [11–18].

## New referrals and outpatient clinics

All of the guidelines recommend minimising face-to-face appointments due to the infective nature of COVID-19 from asymptomatic carriers and the subsequent risk to patients and staff [14, 16–18]. New referrals, therefore, need to be triaged based on the likelihood of detecting a breast cancer and managed accordingly. This can be done by risk stratification, agreed by the clinical team. For example, units severely affected could consider only offering patients under the age of 25 a telephone consultation unless identified as high risk, as the rate of breast cancer in patients this age group in the UK is 1.5 in 100,000 per year [14, 19]. Remotely assessing patients in residential or nursing homes, especially those with multiple comorbidities may also be appropriate, with the consideration of empirical endocrine therapy where a malignancy is felt likely by the referring physician. With all patients where practice deviates from that of the normal unit protocols, the patient’s details should be entered into a database to allow appropriate delayed follow up once safe to do so and for audit purposes. It is also critical that units review their guidelines regularly with regards to local COVID-19 infection rates [14, 18].

It is likely that COVID-19 may alter some outpatient practices forever. For example, patients presenting with isolated breast pain from now on may be offered a telephone consultation only to ensure there are no other symptoms which require a clinical review. They could then be discharged with safety netting advice, as current evidence suggests their risk of breast cancer is equivalent to that of the screening population [20].

Social distancing will probably be in place for a considerable period of time, and therefore units need to consider how to manage the flow in the outpatient department to promote this. Patients should be informed to arrive at the time of their appointment and waiting rooms should be arranged to allow 2 m between patients. Units could consider patients being sent directly to imaging and discharging at this point if no concerns have been identified or then sent onto the breast clinician if there are continued concerns or a biopsy takes place. It needs to be clearly documented in the letter to the patient’s usual physician that the patient was not examined, and patients should be given information regarding how to access the service if they have ongoing concerns, or worsening of symptoms. If an abnormality has been identified, and biopsy taken, ABS guidelines recommend a clip should be placed at the same time to reduce requirement for further attendances [14, 16, 17].

Telemedicine has become a more widely used tool during the COVID-19 pandemic, and may well be utilised further as clinicians and patients become accustomed to it. Follow up appointments where possible should be conducted in this manner, and ESMO and CPBCC suggest that it should be used for new diagnosis of non-invasive cancers [16, 17]. As face-to-face contact with a patient is likely to remain a limited resource, appointments need to be used effectively; for example, in it should be conveyed to patients if there is a high index of suspicion of breast cancer on triple assessment at the initial appointment [14, 16, 17]. This then gives the patient time to process the information prior to the next appointment, so that the most value can be had by both the patient and the clinician from the subsequent appointment.

Appropriate personal protective equipment (PPE) needs to be worn by all staff in clinic. As per the World Health Organisation (WHO) Public Health England (PHE), and ABS guidelines, this should include a fluid resistant surgical mask, gloves, apron and eye protection [21–23].

## Operative decision making

ABS, CPBCC, the Academy of Royal Medical Colleges and ESMO guidelines all agree that prioritisation decisions may need to be made with regards on who to operate. Planning for lists needs to be forward looking, appreciating that it is challenging to predict the demand for resources such as ventilators and theatre space, but equally acknowledging that wherever possible operating needs to continue to ensure patient safety [12, 14–18].

All of the guidelines acknowledge that patients who have recently completed neoadjuvant chemotherapy should be prioritised for surgical management of their breast cancer. Other key patient groups who should be prioritised are those with oestrogen receptor (ER) negative disease, human epidermal growth receptor 2 (HER2) positive disease and those who are pregnant, to attempt to avoid chemotherapy whilst the demand of COVID-19 is at its highest [12, 14, 16–18].

There is some debate, however, between the guidelines, regarding patients who are ER positive and HER2 negative. The CPBCC suggest that only patients who are T1N0, regardless of menopausal status are appropriate for primary endocrine therapy [17], whereas ESMO suggests it is appropriate for those with stage 1 or 2 breast cancer, which would include T2N1 disease, as long as it is low grade, and with consideration of menopausal status [16]. The ABS and Academy of Medical Royal Colleges focus more on menopausal status, and suggest that if surgical access is limited, pre-menopausal patients should be a priority, and post-menopausal patients should be started on primary endocrine therapy with the aim for deferred surgery [12, 14, 18]. This advice reflects the limited evidence base for primary endocrine use in pre-menopausal patients as opposed to post-menopausal patients [24, 25]. Interestingly, studies have looked at using primary endocrine therapy in post-menopausal patients with stage 2 and 3 breast cancer, and seen positive results, suggesting that limiting this approach to T1N0 patients may not be appropriate if theatre space is in demand [26–28].

CPBCC suggests deferring surgery for all ductal carcinoma in situ (DCIS) if there are any concerns regarding hospital resources in the ensuing weeks, and ESMO and ABS rank it as the lowest priority for surgical management, with the exception of “extended high grade change”. Consideration may be given to testing DCIS for ER positivity and starting endocrine therapy if appropriate[14, 16–18].

All of the guidelines agree that operations for benign disease, B3 excision biopsies, delayed reconstruction and symmetrisation should be deferred at present. ABS does not recommend performing immediate reconstructions at present, and ESMO puts them as low priority. (14,16,18) The CPBCC suggests only performing implant or tissue expander based reconstructions, however if resources are limited, then priority must be given to oncological management [17]. It also necessary to recognise that complication rates, including re-admissions, following immediate reconstruction, are significantly higher than a simple mastectomy, and therefore may be exposing patients to increased risk of COVID-19 transmission [29].

Some units have continued to operate on most patients with breast cancer during the COVID-19 pandemic, utilising varying techniques to keep patients safe. It is important to try and perform most operations as day cases, to minimise inpatient stays, reducing potential transmission of COVID-19. Use of local anaesthetics, reduction of intra- and post-operative opiates, and minimising the use of drains wherever possible can be helpful in promoting day case surgery. It also needs to be communicated clearly with the patient, to ensure an appropriate expectation of their hospital experience [30]. Units have also utilised other resources, including local private hospitals, to create centres which are dealing with planned elective cases only. More recently, the Royal College of Surgeons have introduced guidelines for the re-introduction of elective surgery. These involve patients self-isolating for 14 days pre-operatively, being screened for COVID-19 2 days pre-operatively, and units identifying ‘green areas’ in hospital, where only planned elective surgical patients are managed [31]. Implementing this strategy does however require capacity for testing, and it should be noted that the sensitivity for the COVID-19 reverse transcriptase polymerase chain reaction test is around 80%, and therefore a negative test needs to be interpreted in the context of any symptoms [32].

Communication with patients is extremely important in this situation with regards to risk, both from deferral of surgery, and from admission to hospital during the pandemic. MDT documentation should include the standard option, and whether there is another appropriate option given the COVID-19 pandemic. It is also again critical to record all patients who received ‘non-standard’ treatment, and ensure they are followed up appropriately.

## Practical considerations for operating

There has been conflicting information given regarding PPE to be worn by surgeons. The main defining factor is whether an aerosol generating procedure (AGP) is being performed which include intubation, laparoscopy and use of high energy devices. The primary concern is large volume aerosolization of SARS-CoV-2, which could be harmful to staff in theatres [7, 9, 33, 34]. A recent meta-analysis found that standard surgical masks were not inferior to N95 respirators (similar to FFP3 masks), when performing non AGPs in patients with viral respiratory illnesses, and therefore FFP3 masks should be reserved for AGPs only [9].

Current practice, as advised by WHO and PHE, involves the anaesthetist and their assistant wearing full PPE (long sleeved gown, FFP3 mask, double gloves, visor and hood) for intubation [21, 22]. The theatre should be otherwise empty of staff at this point, with minimal equipment in theatre as possible. The evidence has demonstrated that once a space has undergone five air changes then then less than 1% of aerosolised particles are likely to be present. Therefore, as the rate of air changes is approximately 15–20 times per hour in operating theatres, the whole team can then enter wearing standard PPE (surgical mask, gown, gloves and visor) after 20 minutes. Obviously, as laminar flow increases, the rate of air change, this time is markedly decreased. This process is repeated for extubation [33–35]. This process adds to the anaesthetic time required, and this should be factored in when booking lists, especially as the anaesthetic team will need a rehydration break between every case.

Breast surgery has not been specifically designated as an AGP, however, diathermy is used, and there should be some safety consideration with regards to this. Small studies have shown than viral DNA may be present in diathermy plume, but the infectivity to staff remains unknown [36, 37]. The quantity of SARS-CoV-2 that could be generated by this process in breast surgery also remains unclear. What is apparent, however, is that the usage of smoke extraction devices significantly reduces the amount of smoke reaching the surgeon’s mask, and therefore could help reduce any potential risk of transmission [38]. Even with the introduction of pre-operative self-isolation and screening, it is likely that these practical considerations for theatre will remain relevant, primarily for the protection of theatre staff.

## Chemotherapy

The obvious concern regarding chemotherapy is the effect of COVID-19 on the immunosuppressed patient. Small studies from China have shown that cancer patients are more likely to become infected by SARS-CoV-2, and are more likely to require intensive care however at present the data is very limited [8, 39]. Both ESMO and CPBCC state that decisions regarding neo-adjuvant chemotherapy should be taken in the light of current institutional resources, whereas ABS suggests it should be reserved at present for patients with inflammatory breast cancer and non-operable breast cancer [14, 16, 17]. This is in line with the recommendations regarding COVID-19 and chemotherapy from National Institute of Clinical Excellence (NICE), who state that priority should be given to patients where chemotherapy would give a ‘greater than 50% chance of cure’ when compared to standard treatments alone [13]. Long-term data from the Netherlands have shown that the introduction of neoadjuvant chemotherapy for patients with inflammatory breast cancer has helped in a dramatic improvement in 5 year survival; from 17.2% in the early 1990s, to 38.9% in 2015, and therefore is still required in this patient group despite risks from COVID-19 [40]. In patients already started on neo-adjuvant therapy, it may be useful to consider finishing chemotherapy mid treatment if response has either been very good, or conversely minimal, to avoid unnecessary immunosuppression.

Adjuvant chemotherapy should also be discussed on a case by case basis, looking at co-morbidities, risk from COVID-19, and predicted survival benefits [12, 14, 16, 17]. The studies have shown that adjuvant chemotherapy can be delayed in early breast cancer, without adverse outcomes for up to 12 weeks, which may be beneficial for patients in units with a current high COVID-19 infection rates [41]. Consideration should also be given to the increasing the use of genomic testing such as OncotypeDx, to identify patients who are most likely to benefit from chemotherapy, especially in borderline cases, such as patients with 1–3 lymph nodes positive. Interim results from the OncotypeDx node positive trial were presented at the San Antonio Breast Cancer Symposium in 2019, and demonstrated that 74% of ER positive, HER2 negative early breast cancer patients with 1–3 nodes lymph nodes positive avoided chemotherapy following OncotypeDx testing [42]. It is also important to bear in mind, that unlike in haematological malignancies, immunosuppression from chemotherapy for breast cancer is usually transient and may be managed using colony stimulating factors, and therefore the likelihood of disease progression or recurrence needs to be balanced against the risks from COVID-19 [43].

For patients with HER2 positive breast cancer, consideration can be given to adjusting their therapy to help mitigate the risks from COVID-19. Administering trastuzumab sub-cutaneously where possible may avoid unnecessary hospital attendances. For patients with low risk HER2 positive disease, 6 months as opposed to 12 months of adjuvant trastuzumab could be given as per the PHESPHONE trial protocol [44]. In patients with high risk HER2 disease, where there are concerns regarding existing co-morbidities and risk from immunosuppression, the possibility of giving trastuzumab without chemotherapy could be discussed. It should be explained to the patient that disease free survival is reduced with trastuzumab alone, but that risk may be balanced against the possibility of significant issues from infection with COVID-19 [45]. As previously noted, all decisions regarding chemotherapy in the context of COVID-19 must be made in a multi-disciplinary setting, with a good awareness of the COVID-19 infection rates in the local area.

## Radiotherapy

Guidelines specific to radiotherapy for breast cancer in the context of COVID-19 have been developed by experts from Europe, Australasia and the USA [11]. The primary focus is to safely minimise the number of patients who have to come into hospital for radiotherapy, to protect both patients and staff from COVID-19 transmission, and also reduce demand on hospital resources. Both ESMO and CPBCC guidelines also address this issue. They all suggest that radiotherapy could be potentially omitted in patients over 65 years of age, who have ER positive, HER 2 negative, grade 1 or 2 tumours, which are less than 30mm in size, and are taking adjuvant endocrine therapy [11, 16, 17]. This is as per protocol from the PRIME 2 trial, which did not demonstrate any 5 year survival benefit from radiotherapy in this group [46]. The international guidelines do not address radiotherapy in the context of DCIS, but both ESMO and CPBCC guidelines suggest it is a low priority, and could potentially be omitted, especially if the DCIS is ER positive, and the patient is taking adjuvant endocrine therapy.(16,17)

A reduction in the number of fractions that are received to 5, as per the FAST and FAST forward trials, has also been proposed for patients with node negative disease. It is noted that the 5 year local recurrence rates have not been published for FAST forward, however they are expected soon, and the results at 3 years were promising [47, 48]. Avoidance of boost radiotherapy is recommended, unless there are significant risk factors for disease recurrence or in a patient under 40, due to the complications it adds to the planning and administration of radiotherapy [11].

Patients with high risk disease, such as inflammatory breast cancer, triple negative or HER2 positive disease, an incomplete response to neoadjuvant chemotherapy or those under 40 should be high priority for radiotherapy. In these cases, moderate hypofractionation should be used (40Gy in 15 fractions over 3 weeks). It is acknowledged, however, that this is standard practice in many countries [11, 16, 17].

It is possible that some of the changes in how radiotherapy is given, may well become standard practice. As with all aspects of breast cancer care, however, it is important that the risks and benefits of radiotherapy are discussed with the patient, and that deviation from usual protocol is carefully documented. It will be useful to ensure these patients are followed up in the long term, to identify whether these changes in practice, based on established study protocols, have a similar effect when applied pragmatically, as opposed to in a trial setting.

## Conclusion

The effect of the COVID-19 pandemic on the management of early breast cancer is wide ranging, and has forced a change in practice to protect both patients and staff. Minimising face-to-face patient contact is important, however, it is crucial to provide other methods for patients to receive care safely, and standard practice may have to be modified to achieve this. The communication between all involved parties has become more important than ever, to explain complex concepts of risk and benefits from every treatment, in relation to COVID-19. Documentation during this period is key, initially to ensure patients whose treatment is deferred are not lost to follow-up. Subsequently, however, identifying patients where management has deviated from that of standard practice will allow monitoring, and will add to our knowledge of early breast cancer, potentially changing future management strategies. Flexibility during the period of reintroduction of services, whilst awaiting a definite management plan for COVID-19, is critical. Units will need to establish a ‘new normal’, balancing breast cancer care, and the risk of COVID-19 transmission, which will be variable depending on the local area’s rate of infections. The long-term effects of the COVID-19 pandemic on early breast cancer care are difficult to predict, but it is clear that our practice is unlikely to be the same again.

## Funding statement

No funding was received for the research or publication of this paper.

## Conflicts of Interest

There are no conflicts of interest from either author.
